# Conditions for Insertion of the Laryngeal Mask Airway in an Innovative Method vs the Classic Method

**DOI:** 10.5812/aapm-140999

**Published:** 2023-12-07

**Authors:** Hamidreza Shetabi, Ali Shahriari, Omid Aghadavoudi

**Affiliations:** 1 Anesthesiology and Critical Care Research Center, Isfahan University of Medical Sciences, Isfahan, Iran

**Keywords:** Laryngeal Mask Airway, Insertion Techniques, Classic Method, Triple Maneuvers

## Abstract

**Background:**

A laryngeal mask airway (LMA) is a simple and non-invasive device used to establish the airway and maintain ventilation and oxygenation during short-duration medical procedures.

**Objectives:**

This study aimed to compare the placement of an LMA using an innovative technique vs the classic method.

**Methods:**

This clinical trial was conducted at Faiz Hospital in Isfahan. Out of 110 candidates for elective eye surgery, 10 patients were excluded from the study as they did not meet the inclusion criteria. Ultimately, 100 patients were randomly allocated to 2 groups of 50 each. One group underwent LMA insertion using the classic method, while the other group underwent insertion using the face-to-face triple maneuver technique (FFTMT). Data, including laryngeal mask insertion conditions, hemodynamic responses, and clinical complications, were collected, entered into SPSS version 26, and analyzed.

**Results:**

There were no significant differences between the 2 groups in terms of laryngeal mask placement time (P = 0.061), number of attempts to place the LMA (P = 0.059), oropharyngeal leak pressure (OLP; P = 0.942), frequency of hoarseness (P > 0.99), or laryngospasm (P > 0.99). However, it is noteworthy that FFTMT appeared to provide easier placement of the LMA (P < 0.0001), required fewer attempts, and offered better quality of ventilation with a lower presence of blood on the cuff (P = 0.038). Conversely, the FFTMT group had a higher frequency of sore throat (P < 0.0001).

**Conclusions:**

The performance of LMA using FFTMT is comparable to the classic method. In procedures under general anesthesia where the surgeon has access to the patient’s head and neck (such as cataract surgery), airway management with LMA using FFTMT (while maintaining the patient’s sterile covering) appears to be effective in case of an emergency.

## 1. Background

Ensuring safe airway access and adequate ventilation are essential responsibilities of an anesthesiologist during general anesthesia and patient cardiopulmonary resuscitation (CPR) (1). One of the most significant advancements in airway management over the past 35 years has been the invention of the laryngeal mask. Dr Archie Brain invented the laryngeal mask in 1988, and its clinical use has since expanded (2). The laryngeal mask is a simple and non-invasive device used to establish an airway and maintain ventilation and oxygenation during various surgical procedures, including elective and short-duration surgeries, as well as emergency situations like CPR and difficult intubation. Compared to laryngoscopy, the laryngeal mask has fewer adverse effects and causes fewer hemodynamic changes (3).

A classic laryngeal mask consists of an oval silicone mask with an inflatable cuff, which is placed in the hypopharynx and occupies the peri-glottic space. This device also has a tube connected to the ventilation circuit. Proper placement of the laryngeal mask depends on various factors, including the patient’s airway anatomy, obesity, mask size, skill and experience of the anesthesiologist, and the patient’s position (4). The use of a laryngeal mask during anesthesia can help prevent high blood pressure and tachycardia because of hemodynamic stability (5).

According to the classic method developed by Dr Brain, most anesthesiologists stand over the patient’s head when placing the laryngeal mask. Goyal et al. introduced an alternative technique for laryngeal mask insertion, where the anesthesiologist stands beside the patient and uses their thumb (thumb insertion). Their study showed that both groups were comparable in all aspects, with thumb insertion proving to be an effective method for laryngeal mask airway (LMA) insertion (6).

Eglen et al. conducted a study comparing 3 laryngeal mask placement methods: The classic method (Dr Brain), the rotation method, and the triple maneuver (opening the mouth, head extension, and jaw thrust). The results indicated that the time required for successful insertion was significantly shorter with the triple maneuver method compared to the other 2 methods. However, there were no significant differences in variables such as bleeding, sore throat, and placement success on the first attempt (7).

Additionally, Fard and Akhondi compared the classic method with an alternative approach that involved using the nondominant hand to prevent the cuff from contacting the soft palate. Their findings showed that the sore throat rate was 7.5% in the classic method, while the alternative method resulted in a rate of 1.5% (8).

In the classic method and the other proposed techniques, the placement of the mask is typically carried out from above the patient’s head. Only 1 method, known as the thumb insertion technique, involves placing the mask from the patient’s side using the thumb (6). It is worth noting that in various scenarios, such as when a stereotactic frame is placed on the patient’s head or in cases of traffic accidents and natural disasters, where being above the patient’s head may be impractical or impossible, the speed of rescue becomes the top priority. Therefore, the development of innovative methods for LMA placement is particularly important when conventional methods may not be successful (7).

Hashemi et al. evaluated 257 patients and assessed 4 techniques to administer LMA. These techniques included the standard method, mask placement with a 90° rotation, mask placement with a 180° rotation, and the thumb insertion method. Their findings indicated that the 90° rotation method had a significantly higher success rate and a lower failure rate for successful mask placement compared to the other 3 methods. However, no significant differences were reported between the 4 groups of patients regarding their initial vital signs (9).

Aghdasi et al. conducted a study comparing the success rate of LMA insertion using the classic and rotatory methods in pediatric patients undergoing general anesthesia. Their conclusion was that both insertion techniques worked effectively in pediatric surgical patients, with comparable success rates and complications between the 2 groups (10).

In a study conducted by Haghighi et al., 100 orthopedic patients were divided into 2 groups, each consisting of 50 individuals. These groups were subjected to the placement of a laryngeal mask using either the classic or airway method. In the classic method, the index finger was used as a guide to push the back of the LMA against the hard palate, entering the pharynx until resistance was felt, and then fixing the LMA in place. Conversely, the airway method involved inserting the LMA into the mouth at a 180° angle from the inside to the outside, without the use of a finger, until it reached the pharynx (sudden loss of resistance), and then returning it to its natural position before fixing it in place. The researchers concluded that the airway method is a simple and preferable technique with fewer complications for LMA placement (11).

Shyam and Selvaraj used 3 distinct LMA embedding methods. The first method involved the standard technique, whereby the LMA was inserted through the conventional digital intraoral approach. The second method, known as the 90° rotation technique, entailed the counterclockwise rotation of the LMA to 90° within the oral cavity, followed by its advancement until encountering hypopharyngeal resistance, and then flattening it in the hypopharynx. The third method, referred to as the 180° rotation technique, involved the insertion of the LMA from the back to the front, similar to Godel’s airway. Based on their findings, the researchers concluded that the 180° rotation technique of LMA insertion is more effective than the 90° rotation technique in adult patients under general anesthesia (12).

## 2. Objectives

This study aimed to compare the placement of LMA using an innovative method (face-to-face triple maneuver technique [FFTMT]) vs the classic method. The study compared insertion time, ease of insertion, ventilation quality, hemodynamic status, and complications after LMA insertion between the 2 groups.

## 3. Methods

This clinical trial was a double-blind, randomized study involving elective surgery candidates undergoing general anesthesia with LMA placement. The study was conducted from 2021 to 2022 at Faiz Hospital, affiliated with Isfahan University of Medical Sciences. The study was approved by the Research Ethics Committee of Isfahan University of Medical Sciences (code: IR.MUI.MED.REC.1400.074); it was also registered in the Iranian Registry of Clinical Trials (code: IRCT20200217046523N15). 

### 3.1. Inclusion Criteria

Patients meeting the following criteria were included in the study: American Society of Anesthesiologists (ASA) class I and class II, aged 20 to 80 years, weight between 40 and 80 kg, body mass index (BMI) less than 35, mouth opening greater than 25 mm, surgery duration less than 2 h, (7-9) and providing informed consent to participate in the study.

### 3.2. Exclusion Criteria

Patients meeting any of the following criteria were excluded from the study: Mouth opening less than 25 mm, Malampati grade above 2, requiring more than 2 attempts to insert LMA, limited neck extension, cervical spine abnormalities, pregnancy, supra-glottic anatomical abnormalities, surgery duration exceeding 2 h, risk of aspiration, need for oral and nasal surgery, and presence of sore throat and dysphagia. The sample size was determined based on the sample size formula of 45 patients in each group with a significance level of 5% (Z_1-α/2 _= 1.96) and statistical power of 80% (Z_1-β _= 0.84). To detect a standardized effect size of D = 0.60 and to account for patient attrition during the follow-up period, we included 55 patients in each group.

This investigation was conducted in a double-blind manner, with both the patient and anesthesiologist (who was responsible for recording the data) being uninformed about the laryngeal mask insertion technique. Additionally, the 4th-year anesthesiology resident responsible for placing the laryngeal mask did not participate in data collection.

Randomization of the study was achieved using random numbers generated by the Random Allocation software and sealed envelope technique, assigning participants to one of the 2 following groups: Group A (classic method) and group B (FFTMT).

This clinical study was conducted after obtaining permission from the Medical Ethics Committee of Isfahan University of Medical Sciences, with the researcher present in the operating room. A total of 110 patients were assessed for eligibility, but 10 were found to be ineligible because they did not meet the inclusion criteria. Therefore, the study included 100 participants. Subsequently, using randomization software, patients were randomly divided into 2 groups: Classic (50 subjects) and FFTMT (50 subjects) groups. In the classic group, 5 patients were excluded from the study due to the need for more than 2 attempts for LMA insertion, resulting in an analysis being performed on 95 patients.

After obtaining informed consent from all study participants, meticulous records were collected, including demographic and clinical information. Upon admission to the operating room, intraoperative monitoring was conducted, including continuous electrocardiogram (ECG), noninvasive blood pressure (NIBP), oxygen saturation (SpO_2_), end-tidal CO_2_ (EtCO_2_), and airway pressure. Following preoxygenation, anesthesia was induced through the administration of intravenous fentanyl at 2 μg/kg, propofol at 2 mg/kg, and atracurium at 0.3 mg/kg. In the elderly (over 65 years old), the dose of propofol for induction of anesthesia was reduced by 20%.

Patients were ventilated with oxygen via a face mask for 2 min. Then, an experienced 4th-year anesthesiology resident, proficient in both methods of LMA placement, inserted the LMA. Anesthesia was maintained through the administration of isoflurane in oxygen, along with atracurium (initially at a dose of 0.3 mg/kg, followed by 0.1 mg/kg every 30 min). Mechanical ventilation was performed using a volume-controlled and time-cycled method, with a tidal volume set between 5 - 8 mL/kg to ensure that peak inspiratory pressure remained below 20 cm of H_2_O. The frequency of the ventilator was adjusted to maintain EtCO2 levels between 35- and 40-mm Hg, with an inspiratory to expiratory ratio of 1: 2.

Hemodynamic parameters were measured and recorded at baseline (T1) and 1, 3, 5, and 10 min (T2 - T4) after LMA installation. The selection of the LMA size was based on the patient’s body weight, with size 3 chosen for weights less than 50 kg, size 4 for weights between 50 - 70 kg, and size 5 for weights over 70 kg (13, 14). The sniffing position (neck flexion by head elevation and head extension) is commonly used for the insertion of LMA (15).

In the classic method of LMA insertion, the posterior aspect of the cuff was coated with a water-based lubricant. The LMA tube was then grasped between the thumb and index finger of the dominant hand while the nondominant hand extended the patient’s head. The index finger was used to press the tip of the cuff against the hard palate, while the middle finger opened the patient’s mouth. A gradual inward motion was applied, followed by forward advancement of the LMA until encountering some resistance. Finally, the LMA was lowered with the nondominant hand, and the cuff was inflated to the lowest possible volume required to achieve a pressure of 40 - 60 cm H_2_O.

In the FFTMT insertion group, the anesthesiologist stood next to the patient (face-to-face), and the laryngeal mask was inserted, as described below.

**Figure 1.**
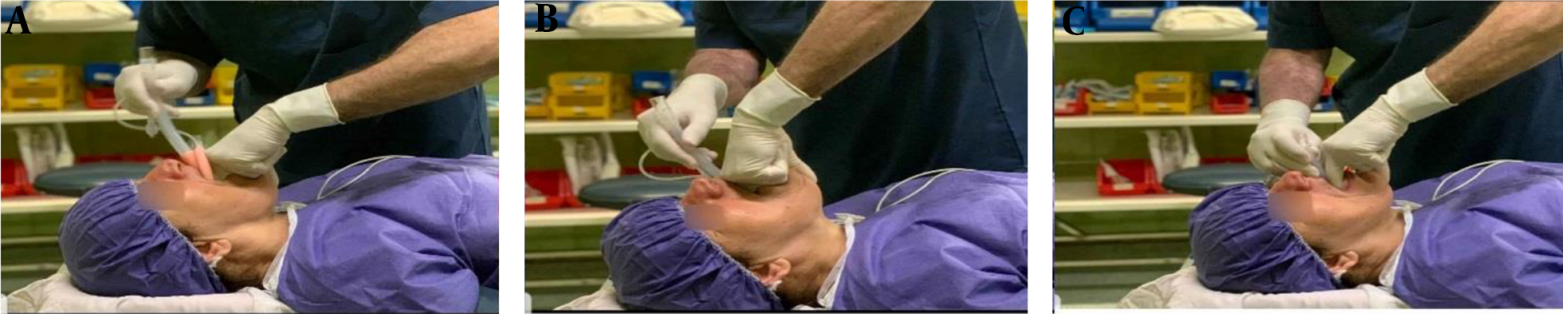
The laryngeal mask airway placement in face-to-face triple maneuvers (face-to-face triple maneuver technique) (A, B, C)

The palmar surface of the second and third fingers of the nondominant hand was pushed forward from the surface of the tongue as far as possible. Then, the Head Tilt-Chin Lift and Jaw Thrust maneuvers were performed (Figure 1). In the next step, using the dominant hand, the lubricated cuff of the laryngeal mask was placed on the fingers and pushed forward until it was in the correct position. Afterward, while holding the LMA with the dominant hand, the fingers of the nondominant hand were removed from the patient’s mouth, and the head position was returned to normal. The LMA cuff was inflated to a pressure of 40 - 60 cm H_2_O, and the LMA was fixed in place.

To ensure the correct placement of the LMA in both procedures, positive pressure ventilation was performed simultaneously with capnography and auscultation.

In this study, an anesthesiologist, who was not part of the research team, assessed and recorded data such as the number of attempts to insert the mask, insertion time (from the moment the anesthesiologist held the mask until viewing the capnograph), the success rate at the first attempt, and the occurrence of adverse events, such as hypoxia, hemodynamic disturbances, presence of blood on the cuff, hoarseness, sore throat, and laryngospasm.

Criteria for successful LMA placement:

1. Establishing a stable airway.

2. Rising of the laryngeal mask when inflating the cuff.

3. The prominence of the anterior part of the neck at the same time as the inflation of the cuff.

4. Placing the laryngeal mask in the central line so that the black line on the posterior surface of the LMA tube is positioned in the central line and aligns with the level of the upper incisors (6).

Criteria for successful ventilation:

1. Achieving sufficient chest expansion.

2. Maintaining stable oxygenation.

3. Displaying square capnography curves.

4. Attaining a minimum tidal volume of 7 mL/kg (6).

If all 4 of the above criteria were met, the ventilation was considered optimal. If any of the above criteria were not met, the ventilation was considered suboptimal.

### 3.3. Evaluation of the Sealing Pressure of Laryngeal Mask Airway or Oropharyngeal Leak Pressure

The oropharyngeal leak pressure (OLP) determines the maximum available airway pressure before air leakage occurs.

To determine OLP, the ventilator was turned off, and the adjustable pressure-limiting (APL) valve was fixed at 30 cm of H_2_O. The fresh gas flow (FGF) was adjusted to 3 L/min. The airway pressure was allowed to increase gradually until it reached a plateau state or the sound of air leakage was heard. At this point, the airway pressure was equal to OLP. A higher OLP indicates better placement and a lower risk of aspiration and stomach distention (16).

### 3.4. Ease of Insertion Criteria

1. Lack of resistance 

2. Mild resistance

3. Moderate resistance 

4. Impossibility of placement (17).

### 3.5. Statistical Analysis

Continuous data were reported as mean ± SD, while categorical data were presented as frequency (percentage). The normality of continuous data was assessed using the Kolmogorov-Smirnov test and Q-Q plot. Quantitative and categorical characteristics of participants were compared between the 2 groups using independent samples t-test and chi-square tests, respectively. Repeated-measures analysis of variance (RM-ANOVA) was used to evaluate changes in hemodynamic variables within and between groups during the follow-up period. The assumption of sphericity in RM-ANOVA was examined using the Mauchly test, and if violated, a multivariate approach was adopted.

The comparison of LMA insertion time, OLP, installation success, ease of insertion, and clinical complications after LMA insertion was conducted between the 2 groups using independent samples *t*-test and chi-square or Fisher’s exact test. All statistical analyses were performed using SPSS version 26 (SPSS Inc, Chicago, IL, USA). A significance level of P < 0.05 was considered statistically significant.

## 4. Results

A total of 110 patients were assessed for eligibility, but 10 of them were found to be ineligible because they did not meet the inclusion criteria. Consequently, this study included 100 participants. These patients were then randomly divided into 2 groups using randomization software: The classic group (50 subjects) and the FFTMT group (50 subjects).

**Figure 2.**
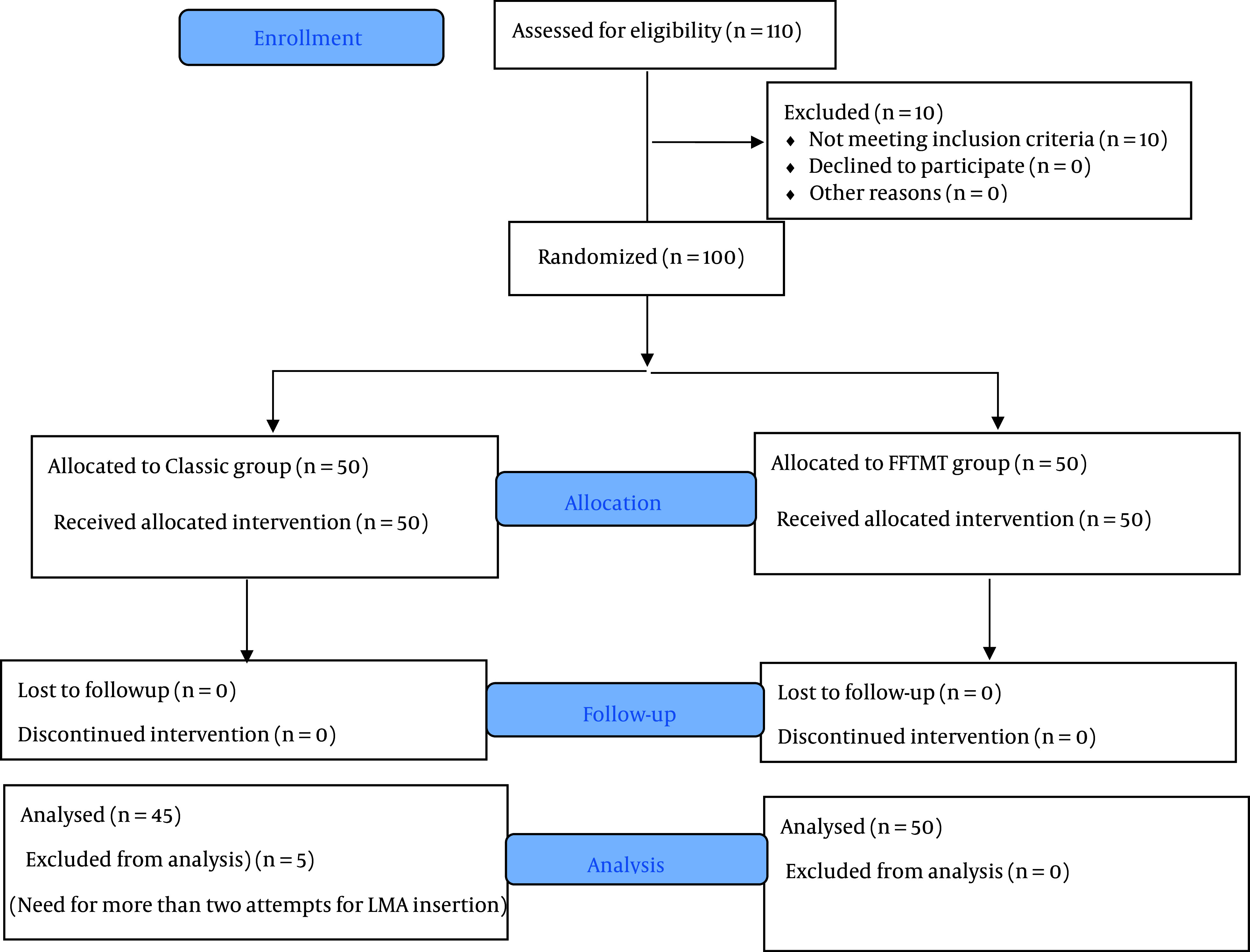
The CONSORT flowchart of patients

In the classic group, 5 patients were subsequently excluded from the study due to the need for more than 2 attempts for LMA insertion. Therefore, the analysis was carried out on 2 groups, with the classic group consisting of 45 patients and the FFTMT group consisting of 50 patients. The CONSORT (Consolidated Standards of Reporting Trials) flowchart of patient enrollment is presented in Figure 2. 

Demographic findings, including age (P = 0.0943), BMI (P = 0.96), gender (P = 0.794), and ASA class (P = 0.06), are given in Table 1. 

**Table 1.** Demographic Variables of Patients in the 2 Groups ^a^

Parameters	Groups		
	Classic	FFTMT	P-Value
**Age (y) ** ^**c**^	58.23 ± 15.68	57.98 ± 17.58	0.0943
**BMI (kg/m** ^**2**^ **) ** ^**c**^	23 ± 2	24 ± 3	0.96
**ASA grade ** ^**d**^			
1	13 (29)	20 (40)	
2	32 (71)	30 (60)	0.06
**Gender ** ^**d**^			
Male	21 (46.7)	22 (44)	
Female	24 (53.3)	28 (56)	0.794

Abbreviations: FFTMT, face-to-face triple maneuver technique; BMI, body mass index; ASA, American Society of Anesthesiologists.

^a^ Values are presented as No. (%) or mean ± SD.

^b^ Statistical test: Student’s *t*-test.

^c^ Chi-square test.

^d^ P < 0.05 was considered significant.

Based on Table 2, it can be observed that there were no significant differences between the 2 groups in terms of laryngeal mask placement time (P = 0.061), number of attempts to place LMA (P = 0.059), OLP (P = 0.942), frequency of hoarseness (P > 0.99), laryngospasm (P > 0.99), and ventilation quality (P = 0.09). However, it is noteworthy that FFTMT showed significantly better ease of LMA placement (P < 0.001) and lower presence of blood on the cuff (P = 0.038). Conversely, the frequency of sore throat was higher in the FFTMT group (P < 0.0001).

**Table 2.** Comparison of Laryngeal Mask Airway Insertion Conditions Between the 2 Groups ^a^

Parameters	Classic Method	FFTMT	P-Value
**Time to insert the laryngeal mask (seconds)** ^**c**^	21.71 ± 6.68	24.32 ± 6.72	0.061
**Number of attempts to insert LMA ** ^**d**^			
1	40 (80)	42 (93.3)	0.059
2	10 (20)	3 (6.7)	
**Ease of Insertion LMA ** ^**d**^			
Lack of resistance	29 (58)	31 (68)	< 0.0001
Mild resistance	21 (42)	14 (32)	
OLP (cm/H2O)^**b**^	22.13 ± 3.75	22.74 ± 3.68	0.942
**Ventilation quality**			
Optimal	44 (97.7)	48 (96)	0.625
Suboptimal	1 (2.3)	2 (4)	
**Presence of blood on the cuff ** ^**c**^			
Yes	5 (12)	1 (2)	0.038
No	40 (88)	49 (98)	
**Sore throat ** ^**d**^			
Low	2 (4.5)	8 (16)	
Moderate	1 (2.5)	1 (2)	
sever	0 (0)	1 (2)	
No	42 (93)	40 (80)	< 0.0001
**Hoarseness ** ^**d**^			
Yes	0	0	> 0.99
No	45	50	
**Laryngospasm ** ^**d**^			
Yes	0	0	> 0.99
No	45	50	

Abbreviation: OLP, oropharyngeal leak pressure.

^a^ Values are presented as No. (%) or mean ± SD.

^b^ Statistical test: Student’s *t*-test.

^c^ Chi-square test.

^d^ P < 0.05 was considered significant.

### 4.1. Hemodynamics

**Figure 3.**
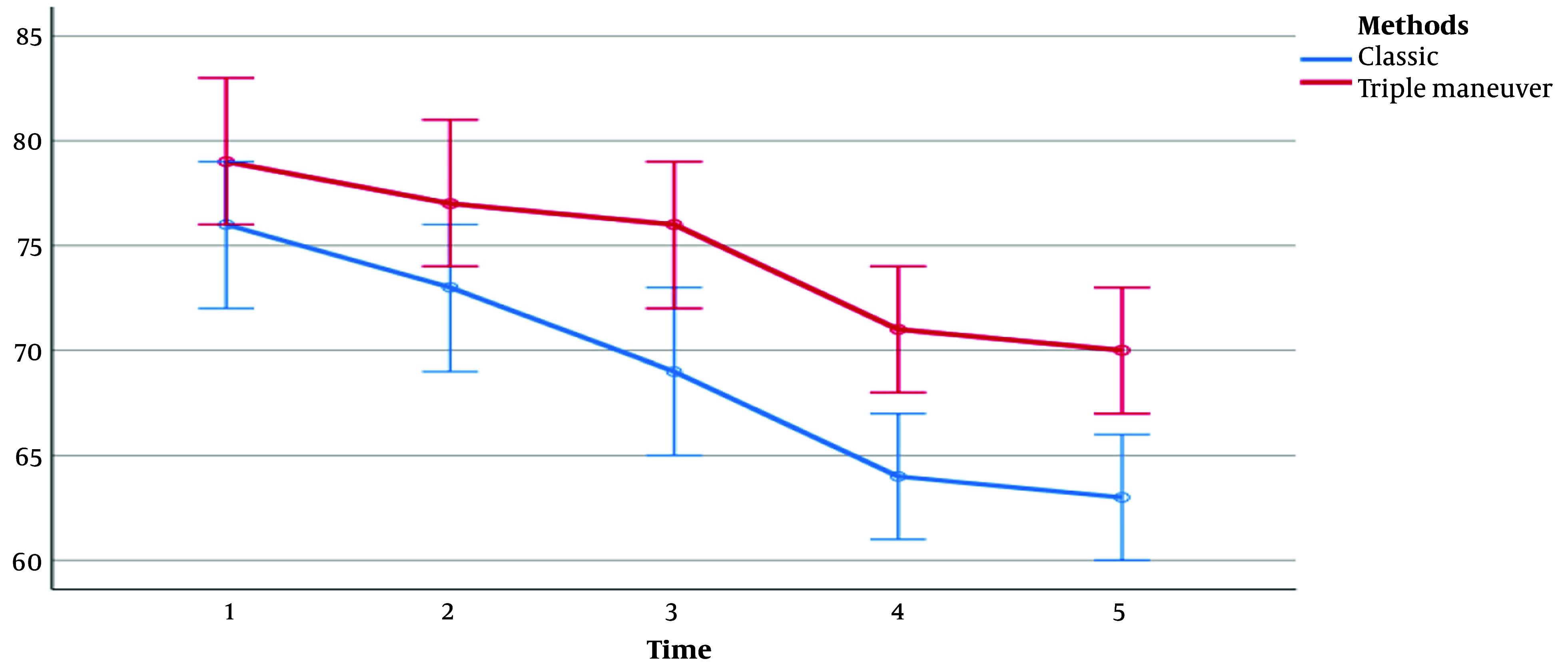
Pulse rate between the 2 groups in 5 periods

**Figure 4.**
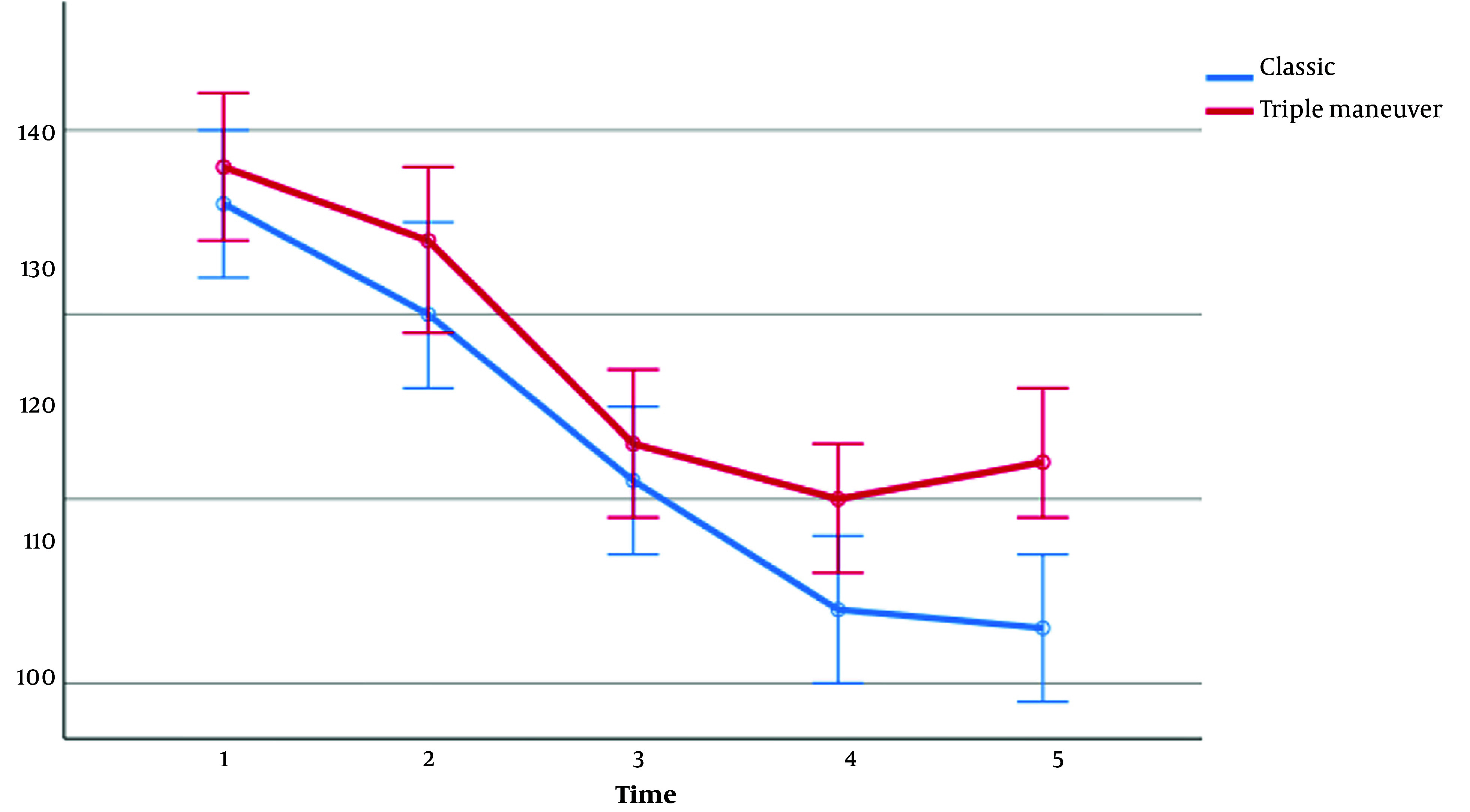
Systolic Blood Pressure between the 2 groups in 5 periods

**Figure 5.**
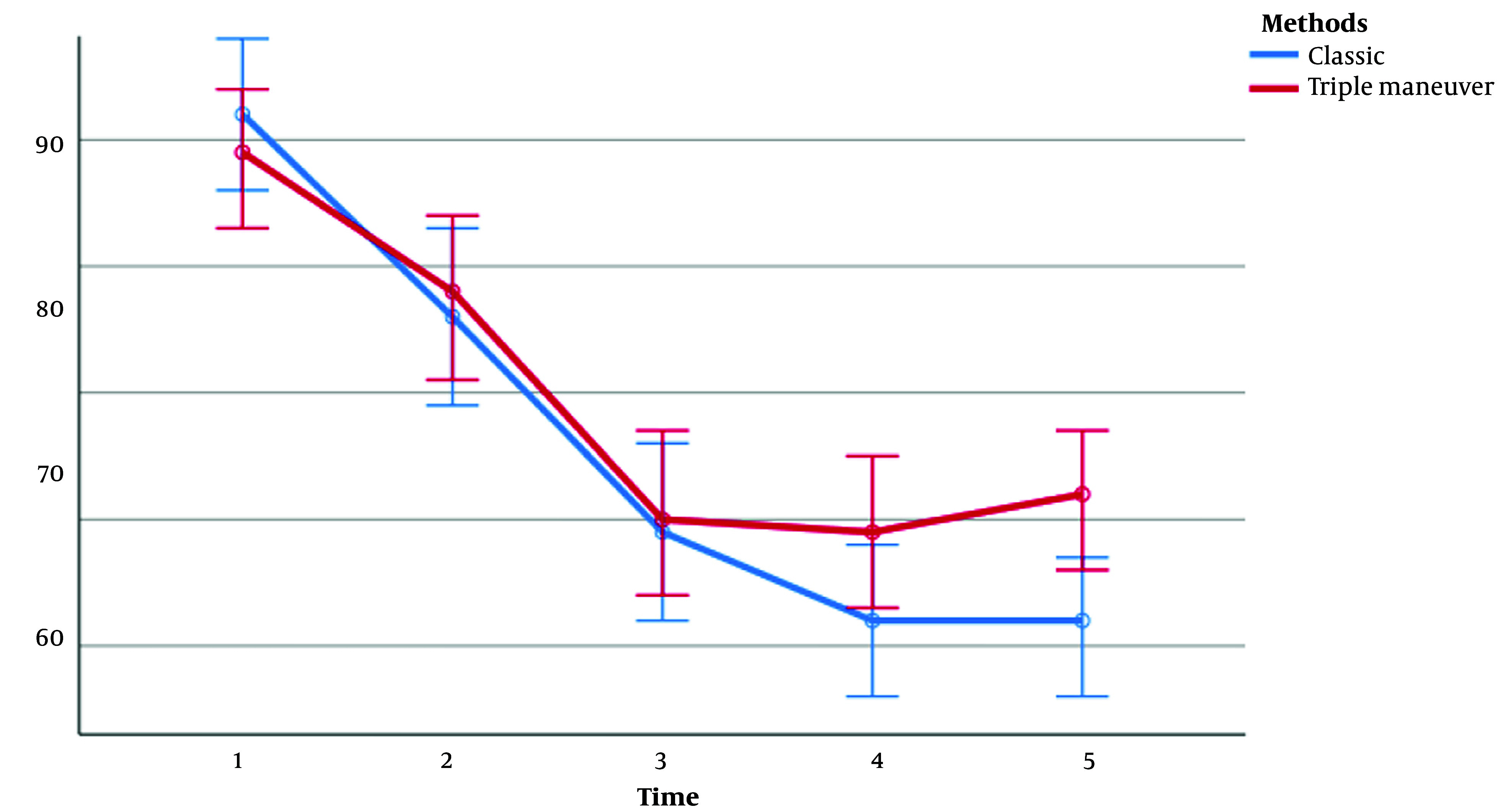
Diastolic Blood pressure between the 2 groups in 5 periods

There was no significant difference between the 2 groups regarding SpO_2_ (during all study periods), heart rate (during T_1_-T_3_ periods), systolic blood pressure (during T_1_-T_4_ periods), diastolic blood pressure (during T_1_-T_3_ periods), and mean arterial pressure (MAP; during T_1_-T_3_ periods). However, during other study periods, the 2 groups showed a significant difference in cardiovascular responses, as evidenced by Table 3 and Figures 3-5. 

**Table 3.** Comparison of Vital Signs Between Patients During Study Times ^a^

Parameters	T_1_, 0 (Initial Time)	T_2_, 1	T_3_, 3	T_4_, 5	T_5_, 10	P-Value, Groups ^b^
**SpO2 **						
Classic	96 ± 3	99 ± 1	99 ± 1.014	99 ± 1	99.09 ± 1	0.280
FFTMT	96 ± 1	99 ± 1	99 ± 0	99 ± 0	99 ± 0	
P-value	0.880	0.211	0.136	0.160	0.167	
**Pulse rate**						
Classic	76.1 ± 11	73 ± 12	69 ± 12	64 ± 10	63.3 ± 9.3	0.006
FFTMT	79.9 ± 13.2	77 ± 11	76 ± 12	71 ± 10	70.3 ± 10.7	
P-value	0.143	0.077	0.009	0.002	0.001	
**Systolic blood pressure**						
classic	142 ± 21	126 ± 22	109 ± 23	102 ± 19	107 ± 14	0.345
FFTMT	139 ± 19	128 ± 23	110 ± 22	109 ± 22	112 ± 22	
P-value	0.406	0.830	0.813	0.090	0.012	
**Diastolic blood pressure**						
classic	86 ± 14	80 ± 16	71 ± 15	64 ± 12	63 ± 11	0.053
FFTMT	88 ± 11	84 ± 14	73 ± 14	70 ± 13	72 ± 15	
P-value	0.472	0.290	0.538	0.035	0.001	
**MAP**						
classic	112 ± 17	97 ± 20	86 ± 19	80 ± 15	78 ± 14	0.064
FFTMT	112 ± 20	104 ± 19.041	89 ± 18	87 ± 17.048	90 ± 19	
P-value	0.836	0.190	0.453	0.030	0.001	

Abbreviations: FFTMT, face-to-face triple maneuver technique; MAP, mean arterial pressure.

^a^ Values are presented as No. (%) or mean ± SD.

^b^ P-values resulted from repeated-measures analysis of variance.

## 5. Discussion

In the present study, a total of 100 patients underwent LMA insertion using the classic method and FFTMT. Our study findings indicate no significant difference between the 2 groups regarding LMA placement time, OLP, number of attempts to insert the LMA, quality of ventilation, frequency of hoarseness, and laryngospasm. However, the presence of blood on the cuff was significantly lower in the FFTMT group. Additionally, the ease of LMA placement was significantly better in the FFTMT group. Nevertheless, the incidence of sore throat was more pronounced in the FFTMT group. The higher frequency of sore throat in the FFTMT method can result from the irritation caused by inserting the second and third fingers into the pharyngeal area while passing the LMA over the fingers.

Regarding SpO_2_, there was no significant difference between the 2 groups at all study times. However, a significant difference was observed for systolic blood pressure at T_5_ and for heart rate, diastolic blood pressure, and MAP at T_4_ and T_5_. The higher blood pressure and heart rate in FFTMT can be due to the stimulation of the pharynx area by the second and third fingers and the simultaneous application of triple maneuvers.

In Merih Eglens’ study (7), a significantly shorter successful insertion time was reported in the triple maneuver groups, but in our study, no significant difference was seen regarding the time for successful insertion between the classic and triple maneuver groups. The sore throat rate was lower in the standard approach, similar to our study. The quality of ventilation was adequate in both studies and both mentioned techniques. Merih Eglens’ study showed no significant difference between the 2 groups regarding the presence of blood on the mask after LMA removal, but in our study, the presence of blood on the mask was significantly lower in FFTMT.

In a study conducted by Monika Goyal et al., 2 methods of LMA insertion, namely the classic method and thumb method, were compared. The researchers reported no significant difference between the insertion time and the number of successful mask insertion attempts. These findings are consistent with the results of the current study. However, in the current study, FFTMT was used instead of the thumb insertion method (6).

In another study by Hashemi et al., 4 techniques for the insertion of LMA were evaluated, including the standard method, placing the mask with 90° and 180° rotation, and the insertion with thumb technique. The researchers concluded that the 90° rotation method had a significantly higher success rate in mask placement than the other 3 methods. However, no significant differences were reported between the 4 groups of patients regarding primary vital signs (9).

In the above study, vital signs were evaluated immediately after LMA insertion. However, the present study assessed vital signs in 5 periods, including the initial time and 4 periods after mask insertion.

In a study conducted by Haghighi et al., 100 orthopedic patients were divided into 2 groups of 50 individuals. The researchers concluded that the airway method is a simple and preferable technique with low complications for LMA placement (11). The results of their study are consistent with the results of the current study in the number of attempts for successful LMA insertion and the presence of blood on LMA. However, despite the current study, insertion time was significantly lower in the airway method compared to the classic method.

Shyam and Selvaraj explored 3 distinct LMA insertion methods. The first method involved the standard technique, the second method, known as the 90° rotation technique, and the third method (referred to as the 180° rotation technique) (12). The researchers concluded that the 180° rotation technique of LMA insertion is more efficacious than the 90° rotation technique in adult patients under general anesthesia. In the 180° rotation technique, insertion time was longer than the classic method, but the success rate in the first attempt and postoperative bleeding were lower than in the classic method, which is consistent with the results of the current study. However, despite the current study, the sore throat rate was lower in the 180° rotation LMA placement technique.

Our study has several limitations. First, the sample size was insufficient to permit generalization of the findings. Second, we did not use a BIS monitor to monitor the depth of anesthesia; instead, we relied on traditional subjective clinical indicators to assess the level of anesthesia. Third, the study was conducted solely at one hospital and exclusively on patients classified as ASA class I and class II.

### 5.1. Conclusions

The performance of LMA using FFTMT is comparable to the classic method. In procedures under sedation where the surgeon has access to the patient’s head and neck (such as cataract surgery), airway management with LMA using FFTMT (while maintaining the patient’s sterile covering) appears to be effective in case of an emergency. As mentioned before, our study had some limitations in terms of generalizability and evaluating the depth of anesthesia, so we suggest conducting similar research in more hospitals and employing a BIS monitor to monitor the depth of anesthesia for more accurate, trustworthy, and generalizable results.

## Data Availability

The dataset presented in the study is available upon request from the corresponding author during submission or after publication. The data are not publicly available due to privacy and implementing further investigations on these data.

## Clinical Trial Registration Code:

IRCT20200217046523N15.
